# Developing a digital training tool to support oncologists in the skill of information-provision: a user centred approach

**DOI:** 10.1186/s12909-020-1985-0

**Published:** 2020-05-01

**Authors:** Sebastiaan M. Stuij, Constance H. C. Drossaert, Nanon H. M. Labrie, Robert L. Hulsman, Marie José Kersten, Sandra van Dulmen, Ellen M. A. Smets, on behalf of the INSTRUCT project group, Hanneke de Haes, Arwen Pieterse, Julia van Weert, Noor Christoph

**Affiliations:** 1 Department of Medical Psychology, Amsterdam University Medical Centers, University of Amsterdam, Amsterdam, The Netherlands; 2 Amsterdam Public Health research institute, Amsterdam, The Netherlands; 3 Cancer Center Amsterdam, Amsterdam, The Netherlands; 4 Department of Psychology, Health and Technology, University of Twente, Enschede, The Netherlands; 5 Athena Institute, Vrije Universiteit Amsterdam, Amsterdam, The Netherlands; 6 Department of Haematology, Amsterdam University Medical Centers, University of Amsterdam, Amsterdam, The Netherlands; 7 LYMMCARE (Lymphoma and Myeloma Center Amsterdam), Amsterdam, The Netherlands; 8 NIVEL (Netherlands institute for health services research), Utrecht, the Netherlands; 9 Department of Primary and Community Care, Radboud Institute for Health Sciences, Radboud university medical center, Nijmegen, the Netherlands; 10 Faculty of Health and Social Sciences, University of South-Eastern Norway, Drammen, Norway

**Keywords:** E-learning, Doctor-patient communication, Communication skills training, Oncology, User-centered design, Communication technology

## Abstract

**Background:**

For patients with cancer, being well informed by their oncologist about treatment options and the implications thereof is highly relevant. Communication skills training (CST) programs have shown to be effective in improving clinicians’ communication skills, yet CSTs are time-consuming, inconvenient to schedule, and costly. Online education enables new ways of accessible learning in a safe and personalised environment.

**Aim and methods:**

We describe the design of a digital CST-tool for information provision skills that meets oncologists’ learning needs. We used the CeHRes Roadmap for user-centred design as a guiding framework. Phase 1 (Contextual Inquiry) involved consultation of the literature and a focus group interview study to uncover the learning needs and training preferences of clinicians’ regarding a digital training for the skill of information-provision. In phase 2 (Value Specification), two multidisciplinary expert panels specified the learning content and format of a digital training. Phase 3 (Design) encompassed an iterative development process, including two user group assessment sessions and 5 individual user sessions in which prototypes were tested. All sessions were recorded and independently analyzed by two researchers.

**Results:**

Based on literature and consultation of the users in the inquiry phase of the development process, and on expert opinion in the value specification phase, relevant (sub) skills and user requirements were defined to consider for the digital training format*.* It was decided to develop a conventional e-learning and a chatbot. Personalization and interactivity were integrated in the prototypes by including features that allow for e.g., choosing text, video or animation; to upload video-recorded consultations to receive peer-feedback; and to consult a communication expert. Results revealed that, overall, participants expressed a willingness to use a digital training tool to acquire information-provision skills. Individual user testing (including junior clinicians), indicated a preference for the chatbot over the e-learning.

**Conclusion:**

We offer a description of extensive development work which was conducted in collaboration with multiple health care professionals to iteratively develop two innovative prototypes of digital tools that would appropriately engage oncologists in learning effective information giving skills. The resulting prototypes were well appreciated and thus provide a solid basis for further development and testing.

## Background

For patients with cancer, being well informed by their oncologist about treatment options and the implications thereof is highly relevant. Effective communication, including information-provision, serves the patient’s need to understand (instrumental or cognitive needs) and to be understood (affective or socio-emotional needs) [1]. Consequently, health care providers should always use general instrumental and affective communication strategies [2]. Regarding the affective aspect of information-provision, communication theory highlights information seeking as a likely response to illness related uncertainty [3]. Information may reduce uncertainty that is distressing and tied to anxiety, or increase uncertainty that allows for hope or optimism. From a cognitive perspective, information may lead to a reappraisal of uncertainty [3]. Importantly, uncertainty can only be affected if the information is understood and remembered by patients, i.e. if information-provision supports information processing. For this reason, Linn et al. included instrumental information-provision strategies such as summarizing, categorizing or checking for patients’ understanding in their health communication typology [4].

Indeed, information has been found to reduce patients’ uncertainty and anxiety and provide a sense of control [5–8]. Well informed patients have been found to be more likely to complete their therapy [4]. Moreover, being informed about treatment options, procedures and outcomes in terms of harms and benefits is necessary to involve patients in shared decision making [9]. Information thus contributes to patient autonomy [10]. Hence, information-provision is essential for optimal cancer care and skilled communication is a basic competency for oncologists [11].

Information-provision can be considered more effective when it is tailored to patients’ information needs and level of understanding, is structured and balanced, enhances recall of information, is honest and realistic without destroying hope or creating false hope, and is provided in an empathic way [12]. Thus, information-provision is a highly complex skill which, against clinicians’ own beliefs [13, 14], is not an inborn quality or a natural consequence of professional experience [15], but which needs to be trained. Providing information to cancer patients is particularly challenging due to the complexity of cancer treatment and the strong emotional responses of patients involved. Research shows that information-provision in oncology settings is suboptimal; patients’ information needs are often unmet [16–18], they have trouble understanding clinical terminology [19] and generally remember only a fraction of the provided information, especially when they have low health literacy skills or an older age [20].

Communication skills training (CST) programs have shown to be effective in improving clinicians’ communication skills, including information provision [21], in simulated settings as well as in clinical practice [21–27]. These CSTs commonly involve several face-to-face tutoring sessions in small groups, sometimes with additional booster-sessions [15]. Despite their clear merits, CSTs are time-consuming, inconvenient to schedule, and costly [28, 29]. In recent years, new forms of online education have become available using online interactive and multi-media technologies, including mobile devices. Online education is generally used for knowledge transfer, but it can also be used for skills-based training [28]. Online education enables new ways of learning which are accessible at any time and location, enhancing access to continuous learning for clinicians. Furthermore, it offers learners the opportunity to practice these skills in a safe environment, without direct consequences for patients and without peer-pressure, in their own preferred time and learning environment and allows for immediate feedback and personalized content to meet the individual needs of the learner.

In 2017, we searched the literature for online communication skills trainings. This yielded 13 independent studies published in 2010 or later, describing and evaluating (partly) digital CSTs for health care professionals (*n* = 6) or medical students (*n* = 7) [28, 30–35]. None of the studies involved oncologists (in training). Overall, the digital CSTs were positively evaluated and reported to have educational value. Of these studies, eight reported the effectiveness of such trainings on performance, seven of which demonstrated a learning effect, either by a significant improvement in the experimental group(s) as opposed to a control group [34, 36, 37], or by a significant improvement from pre- to post-assessments [30, 33, 37, 38]. Three training programs addressed information-provision skills, two of which focused on providing bad news rather than treatment information per se [30, 34, 35]. These findings left us to conclude that a digital training tool might be promising for supporting the acquisition of communication skills and that thus far no tool has been developed that could help medical oncologists to communicate treatment information more effectively to their patients.

The aim of this paper is to describe the design of a digital CST-tool for information provision skills that meets specifically oncologists’ learning needs. We developed such a tool, with the aid of the CeHRes Roadmap framework [39]. The CeHRes Roadmap is a user-centred and holistic framework for developing digital health related applications and interventions through iterative steps. It guides effectively the process from idea to successful implementation, by actively engaging the eventual users in the design process. This paper summarizes the development process guided by the CeHRes Roadmap demonstrating the collaboration of our interdisciplinary team with oncologists representing the end-users. By sharing the lessons learned, we provide future researchers and educators with guidance to collaboratively design new digital tools for the acquisition of learning skills.

## Methods

**Fig. 1:**
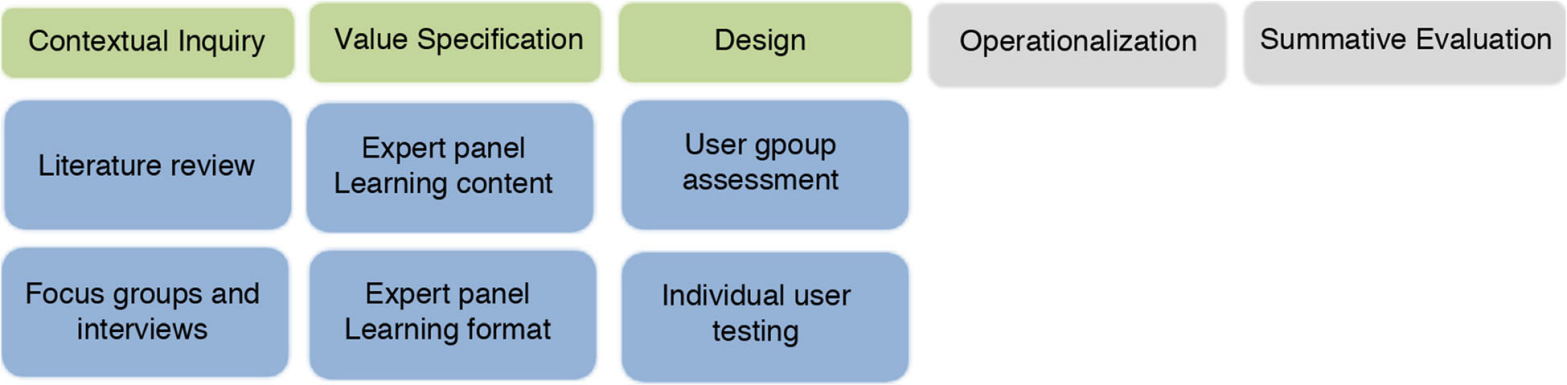
Activities in step 1,2 and 3 of the CeHRes roapmap

The CeHRes Roadmap comprises 5 phases [39]. Briefly, the first phase, called contextual inquiry, entails a needs assessment involving the intended users of the technology and the environment in which it will be implemented. The subsequent value specification phase intends to reveal what should be included in the tool and how it should work, resulting in user requirements. This is followed by a design phase which refers to building prototypes that fit with the user requirements. The operationalization phase addresses the actual introduction, adoption, and employment of the technological intervention in practice. The final evaluation phase refers to the actual use of the technology and the assessment of the impact thereof. This paper describes the activities in the first three steps in the developmental process of the training. Given that the CeHRes Roadmap is meant to be used in an iterative and exploratory way, we conducted several sequential small qualitative studies as initial steps in the development process, with different methodologies for each phase. These are described in greater detail below (see also Fig. 1 for an overview).

One of the qualitative studies, i.e. a focus group study, has previously been published in a separate paper [14]. Here we again, yet, briefly report on this study, to illustrate how the findings of this study fit in the larger design process.

## Results

### Phase 1: contextual inquiry

To uncover the learning needs and training preferences of oncological health care providers regarding a digital training for the skill of information-provision and to gain an understanding of the context, we consulted the literature and conducted an interview study.

#### Literature

To understand the context, we first consulted the literature addressing information-provision skills of oncological health care providers (e.g. studies in which the information giving behaviour of oncologists was addressed, literature on CST, text books on communication in health care). Empirical studies demonstrate that oncologists often provide too much information [40], without checking patients’ information needs [41] and understanding [42, 43]. CST have been found to be effective yet requiring a considerable time investment; they are recommended to be experiential and to last at least 3 days [15], although evidence for optimal training format is still needed [44]. Our review of digital CST, described in the introduction, indicated these to be promising, although few have addressed the specific skill of information-provision. For effective information-provision, instrumental communication strategies such as the ‘teach-back’ and ‘ask-tell-ask’ approach are recommended to respectively check for patients’ understanding and to tailor information to their information needs [45, 46] (see Table 2 for more details about these strategies).

#### Focus group interviews

Using focus group interviews, we aimed to investigate oncologists’ learning needs related to their information-provision skills, and explored their user-preferences about a digital training tool. Full details are presented in a separate paper [14], here we briefly describe the method and main findings.

##### Participants and procedure

To maximise possible variation in needs and preferences, we strived to include participants varying in sociodemographic characteristics (age, gender), clinical experience, from academic as well as general hospitals, and in experience with modern technology. Participants were recruited through a snowball approach using e-mail, phone, and face-to-face meetings. A focus group of 1, 5 h was scheduled per hospital location, including up to 5 people. Separate interviews of approximately 1 h were scheduled with HCP who (eventually) could not attend the focus group session. During focus group sessions and individual interviews, two general domains were discussed: 1) participants’ learning needs concerning information-provision skills, and 2) their training preferences with respect to a new digital training tool on this issue. Finally, perceived barriers and facilitators for a digital training were questioned. A topic guide with open-ended questions was used to guide the interviews’ in a semi-structured fashion.

##### Analysis

All interviews were digitally audio recorded and transcribed verbatim. Thematic content analysis first involved the collective construction of an initial codebook by three researchers (SS, NL, ES) using the topic guide. Transcriptions were then independently analysed and discussed by these researchers until consensus was reached. Subsequently, themes and subthemes were reviewed and refined until researchers agreed that these reflected the essence of the complete dataset. Finally, a summary of the main findings was constructed (SS) and checked (ES), including major themes, noteworthy exceptions, and relevant quotes.

##### Results

Four focus group sessions, with respectively 5, 3, 3, and 2 participants per session (*n* = 13), and three interviews were conducted.

Detailed findings are described elsewhere [14], but it should be highlighted that the most prominent educational needs perceived by the health care professionals included how to 1) tailor their information to the needs of the individual patient, 2) structure and prioritise information during a consultation and 3) deal with patients’ emotions. The oncologists desired a digital training tool to be customized to their individual learning needs, to allow for feedback on their competence and improvement in (recorded) information-provision skills from peers, communication experts, and/or patients and to be able to monitor progress. Additionally, promoting behavior change, i.e., the transfer of skills to clinical consultations, should be a key-feature of such a training. Oncologists in training, rather than senior oncologists, appeared to be most interested in considering digital as opposed to more conventional face-to-face communication skills trainings [14].

### Phase 2: value specification

The value specification phase aimed to uncover which content is important to include in the tool and how the tool should work, based on the learning goals and training preferences of the users as identified in the contextual inquiry phase, and to translate these to concrete user requirements. Our choices were additionally guided by our starting point to adhere to the principles of experiential practice [47] and reflective learning [48, 49] and feedback informed self-assessment [50]. Learning is considered to be an active constructivistic process in which learners discover for themselves the principles that guide effective communication behavior by practicing skills. This process is informed by skills practicing, reflective thinking and peer-feedback [50].

#### Participants and procedure

We set up two multidisciplinary expert panels comprising members of the INSTRUCT project group, who respectively addressed the learning content, and the learning format of the digital training, based on the findings thus far. Both groups met separately, yet exchanged their notes to inform each other. The executive researcher (SS) attended all meetings as a liaison. During meetings, preliminary decisions were made through consensus. At a final meeting, these decisions were critically discussed with the entire INSTRUCT group.

#### Learning content

The learning content*-*panel decided upon which specific skills to address in a first prototype of the digital training tool. This panel included expert members of the INSTRUCT group with a background in communication research in oncology (ES, JvW, AP, RH), and/or communication skills training (ES, RH). Additionally, we consulted an expert in communication skills training of medical specialists (DvW). Building on results of the first phase, which indicated learning needs for tailoring, structuring and dealing with emotions, consensus was reached that the most pressing need of HCP to be addressed in a digital training should be tailoring the amount and content of their information giving to the information needs of individual patients and to take individual patients’ level of understanding, i.e. their health literacy, into account while at the same time keeping control over the structure and duration of the medical consultation. Additionally, professionals’ awareness of specific skills (e.g., ‘teach back’ to check patients’ understanding) yet inability to implement these into practice was believed to be an important focus for a training tool. In subsequent meetings, consensus was reached about the sub-tasks deemed relevant for tailoring of information and the specific skills for these tasks (see Table 1).

**Table 1:** Skills for tailored information provision*

Sub-tasks for tailoring	Skills
1.Estimate of patients’ knowledge level	Use questions such as: e.g. *Can you tell me briefly what you already know about.., so I may try to provide additional information, if needed?*
2. Estimate of patients’ health literacy, intellectual skills	Use Teach-back: Ask the patient to describe in their own words what they understood/ remember from the information provided thus far • “*I gave you quite a lot of information, what would you consider the most important?”* • *“To check whether I have been clear, can you please tell me; what do you remember?”* • *“Shortly you will go home. Suppose your partner asks ‘what did the doctor tell you’?. What will you tell him/her?”*
3. Estimate of and tailoring to patients’ monitoring coping style/ information need	Use questions such as: • *“There is a lot to discuss …*. *Some patients want to have as much information as possible, others prefer not to know too much. How is this for you?”* • *“Will you please tell me if you feel you have had enough information?*” Discuss the outline if the patient indicates a limited need for (additional) information: • *“I will discuss the main points. Should you wish more information on a specific issue, please feel free to interrupt me”* Consider exploring patients’ reason for a limited wish for information (relevant for 8). - If patient indicates a lack of understanding, then adapt information to intellectual level (relevant for 2) - If patient indicates to find additional information too confronting, to provoke too much anxiety, to fear it will reduce hope, then refrain from more detailed information . If the patient appears ambivalent: - Make this ambivalence explicit - probe for clarification - offer the different information topics you can provide (e,g, about treatment procedure, about short and/or long term side-effects, about prognosis Prioritize, if patient expresses a high need for information yet time does not permit extensive discussion, • “*I notice you have many issues to discuss. Unfortunately, we will not be able to discuss these all during this consutation. What would you consider the most important issues that we certainly need to address.”*
4. Assess patients’ stress level to adapt information giving accordingly	Take notice of (non-verbal) signs and make these explicit • *“I notice it all becomes a bit overwhelming for you. Am I correct?*”
5. Assess *which* information the patient finds relevant	Involve the patient in setting the agenda for the consult • *“Are there any issues you would like to discuss now, so we can make the best use of our time?* Ask-tell-ask: Announce an information topic and ask whether the patient is interested (at this point in time), tell, and ask the patient to respond to this information - Examples of an information starters: • *“Would you mind if I tell you something about … …*” • *“What would you like to be informed about?”* - Examples of probes for a response: • *“Is there anything more you’d like to hear about?”* • *“What do you make of this?”* • *“I don’t know what this means to you?”* • *“Is this what you expected?”*
6. Assess how the patient wants to be informed (e.g. by you, by a nurse, in writing, via internet)	At the end of the consultation, ask • *“I gave you a lot of information, would you also like to read about this, for example in a brochure or the internet?*
7. Assess when a patient wants to be informed	Ask for example • *“Would you mind if I tell you something about … …*” • *“Would you like to hear more?”* • *“Is it ok with you if I get back to this the next time we meet? ..* At a follow-up consultation, ask • *“Do you have any questions as a result of what I told you last time?”*
8. Tailor information to the patients’ personal context	Probe for patients pre-existing knowledge (see 1) and his opinion about the information (see 5) Stimulate patients’ question asking: • *“If you have any questions, please let me know.”* Repeatedly invite the patient to share his thoughts, reflections, feelings (see 5): • *“What do you think about what I just told you?”*
9. Check whether your information aligns with patients’ information need.	Ask-tell-ask (see 5), in between your information giving. Do not wait for the end of the consultation • *“Is this enough information for you?”* • *“Which questions do you have?”* • “*Did I forget anything you might want to know?”*
10. Check whether the patients has understood your information	Teach-back (see 2)

*References and sources for these recommendations: Silverman, Kurtz, Draper. Vaardig communiceren in de gezondheidszorg. 2000; Remke van Staveren Patientgericht Communiceren, de tijdstroom, 2013; Back and Arnold, Discussing prognosis, JCO 2006; 24: 4209–4217; C.L. ter Hoeven, L.C. Zandbelt, S. J. Franssen, E.M.A. Smets, F.J. Oort, E.D. Geijsen, C.C.E. Koning, J.C.J.M. de Haes. Measuring cancer patients’ reasons for their information preference: Construction of the Considerations Concerning Cancer Information (CCCI) questionnaire. *Psycho-Oncology*, 2011;20:1228–1235

#### Learning format

The learning format-panel aimed to determine the format for the digital training, and comprised of members of the INSTRUCT group including a user experience researcher (CD), two education professionals (RH, NC), an epidemiologist with a focus on information technology in cancer care (MvO) and an expert in human computer interaction (SS). Three separate group meetings were organised after which individual appointments were made to refine the proposed formats of the digital training tool. In these meetings, the results of the contextual inquiry phase with respect to the professionals’ desired training format were interpreted and translated to user requirements (see Table 2).

**Table 2:** User requirements

User Need	Tool Requirement
**Learning content**	
To understand the importance of effective information provision	1) Offer a short introduction which explains the importance of this topic and training and what the user will gain from it.
To learn effective communication techniques to tailor information to patient characteristics, to structure information giving and how to deal with patients’ emotions as a result of information such as anger and sadness.	2) Offer specific chapters on each topic (tailoring, structuring and dealing with emotions), offering explanation, (video) examples and assignments of effective communication techniques. Tailoring has priority.
**Learning format**	
To feel engaged while learning new skills during training.	3) Offer engaging and relevant content with different types of media: text, video, animations, illustrations and interactive assignments.
To learn new communication skills in a constrained amount of time.	4) It should be as time effective as possible by including personalisation options, 5) avoiding large amounts of text, offering short animations clips, and 6) ending chapters with a summary and take-away message.
Learning from evidence-based content.	7) Include learning content which is concise and built upon scientific literature and include easily accessible links to these sources.
A learning experience tailored to degree of experience and individual learning needs.	8) Offer a pre-test to determine the user’s experience and learning goals. 9) Have the tool generate a personalized training advice.
A learning experience tailored to preferred style of learning.	10) Offer the user different media options with the same learning content.
Examples of desired communication skills	11) Offer video content, preferably recording of real consultations, demonstrating desired communication skills.
Opportunity to practice communication skills.	12) It should allow for practising skills within the learning environment and 13) in daily practice.
Peer feedback on communication skills.	14) It should offer functionality to record a consultation and allow for playback and 15) evaluation of performance by peers
To evaluate progress on communication skills before and after the training.	16) Offer a pre- and post-test and 17) progress insights using a scoring system and/or graph.
To be reminded of newly learned skills in daily practice during training period.	18) Offer notifications and tips aligned to individual learning goals.
Earning accreditation points for training.	19) Emphasize that the training will include accreditation points and 20) offer a certificate of completion at the end of the training.
To be able to consult an expert on communication skills.	21) Include a feature in which the user can contact an expert to ask for feedback and/or make an appointment.

Results of the focus groups indicated that oncologists are increasingly familiar and comfortable with relatively conventional interfaces such as medical e-learning modules, in which a user opens a web-based e-learning application and is presented with learning content in the form of informative text, illustration, video examples and interactive assignments including the use of video annotations. The development of a conventional e-learning was considered to be a viable option, since an e-learning can be tailored to learners’ previous experience, individual learning needs and/or learning style, thereby accommodating HCPs need for personalisation. Moreover, e-learnings have been found to be promising in the context of communication skills trainings [34, 36, 37].

A second idea emerging in the expert discussions encompassed the use of a chatbot, also known as a form of a conversational agent, which refers to an automated digital avatar which interacts with the user through different instant messaging services (e.g. Facebook, Whatsapp) [51].

Conversational agents are increasingly used in the domain of communications skills training, including the domain of communication skills in health. To illustrate, in our preparatory review of the literature on online communication skills training we found three studies involving the evaluation of virtual patients to teach communication skills to medical students [31, 37, 52]. A recent systematic review on the effectiveness of online training for medical students [53], reported on one more study [54]. In all these studies, conversational agents are used as virtual patients. However, the chatbot we envisioned for our training module aimed to guide the learner, i.e., oncologist, through the learning process by taking on the role of coach rather than patient. For example by asking questions via instant messages such as: “Let’s continue with our learning goal shall we?”; “Do you have time for this now?” (yes/no); “Because you indicated a preference for scientific literature, would you like to read a recent article on tailoring I highly recommend?” (yes/no).

The ‘personalized’ approach offered by the conversational interface of a chatbot was assumed to address oncologists’ need for a personalized training program including individualized feedback by creating a highly engaging, personal, and focused, learning experience in which the chatbot acts as a ‘learning coach’. By offering brief, interactive, and location independent learning experiences on their smartphone, a chatbot may accommodate oncologists’ time constraints. To the best of our knowledge, chatbots have not previously been used for CST.

Lastly, to address the need to facilitate transfer of skills to clinical consultations we aimed for a marked alternative for the pocket–sized cards often used by clinicians as a guideline for guidelines and procedures. The use of physical gadgets (e.g., a button, coloured wristband, keychain or badge) as reminders was suggested (inspired by ‘TinyTasks’ by the Technical University Delft [55]). For example, a wristband may serve as a reminder to try and tailor information to individual patients during the day. Physical reminders of specific tasks could be linked to particular digital learning content and as such could be combined with a digital training tool.

Based on the aforementioned arguments, two format ideas for early prototypes of a digital tool were drawn up: a relatively conventional web-based e-learning module and a more innovative chatbot. Additionally, prototypes of physical reminders were to be developed as an add-on to aid learning and skill transfer.

As a final step in the value clarification phase, the results of both the learning content and learning format panels were presented at a plenary meeting of the INSTRUCT project group for feedback and suggestions. Following discussion it was decided 1) to – initially - develop and evaluate separate prototypes of an e-learning, a chatbot and physical reminders, and 2) to continue testing with oncologists in training, because of their greater interest in the possibilities offered by a digital training tool.

### Phase 3: design

#### User group evaluations of three prototypes

Prototypes of increasing fidelity of the two training tools, the conventional e-learning and the chatbot, were developed in an iterative process of prototype design and two user group assessment sessions.

##### Participants and procedure

Feedback on the appreciation of the tools in general and of specific features in particular was gathered from six hemato-oncologists from the department of hematology of the Amsterdam University Medical Center/ University of Amsterdam. They had consented to participate upon invitation by the hemato-oncologist from the INSTRUCT group (MK). Two user group assessment sessions were organized, involving respectively four (3 males) and two participants (1 male).

**Fig. 2:**
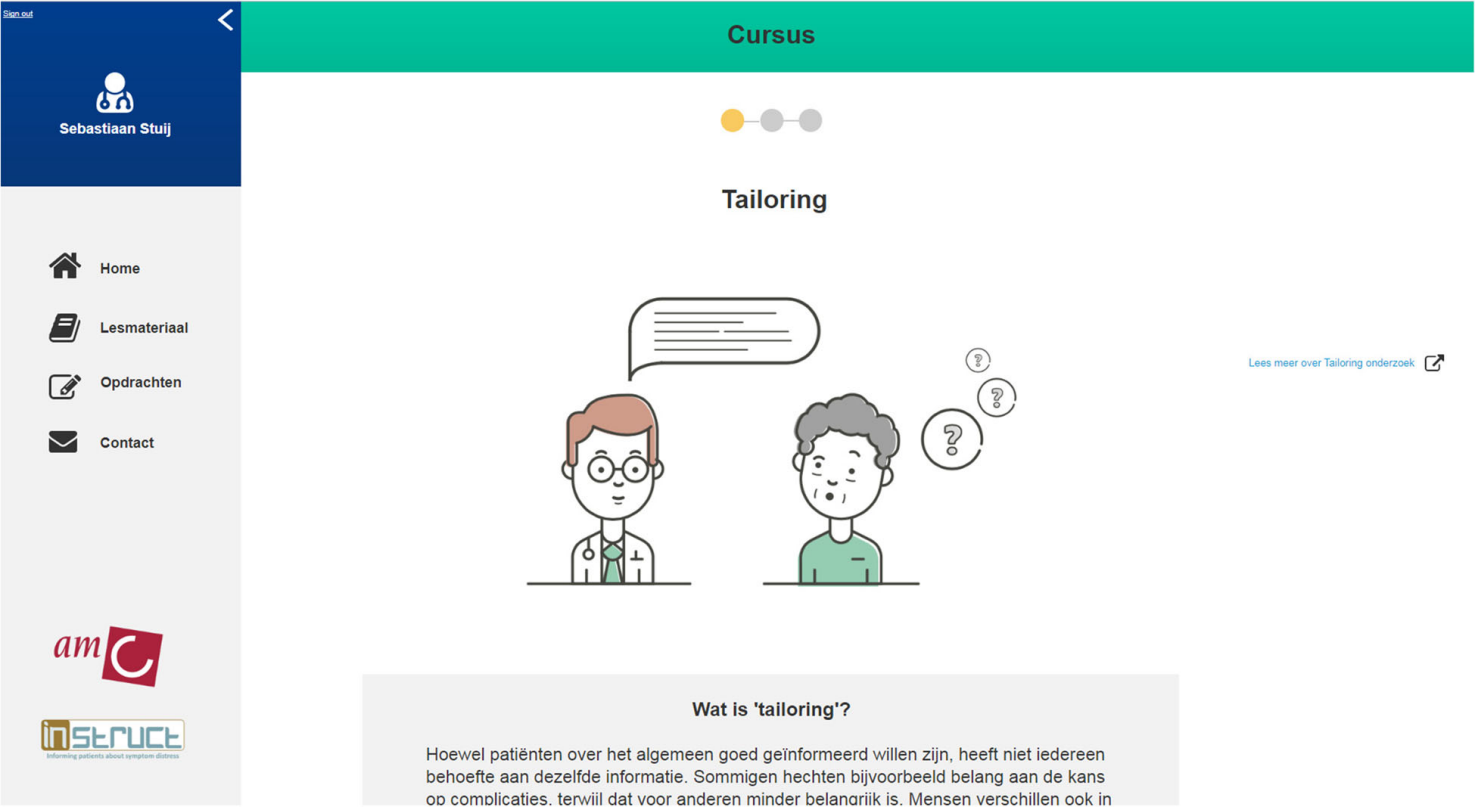
Screenshot of a mock-up of the web-based e-learning tool (created with ‘Axure’ [https://www.axure.com/])

**Fig. 3:**
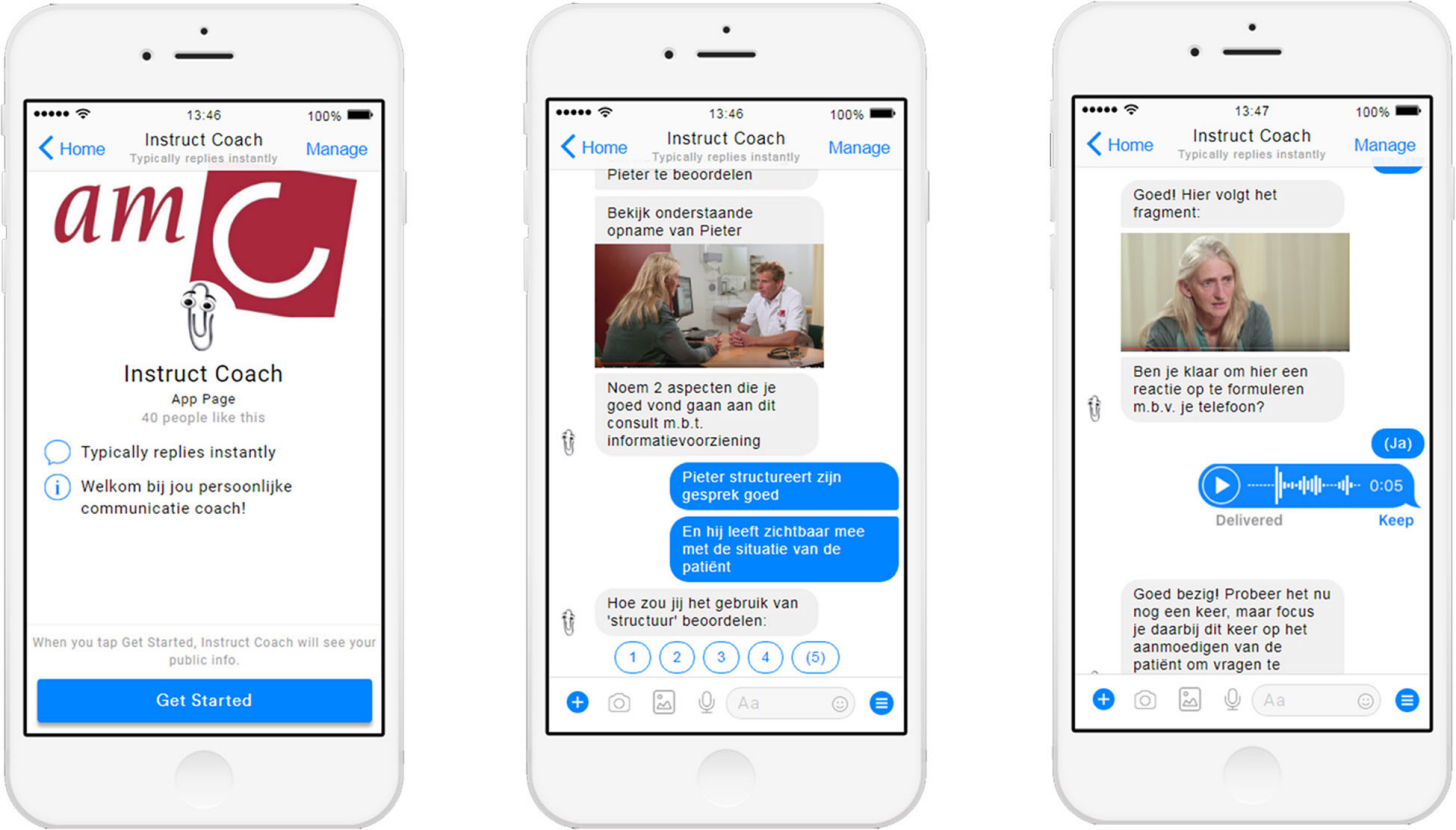
Some screenshots of the mock-up of the chatbot (created with ‘Botsociety’ [(https://app.botsociety.io/])

**Fig. 4:**
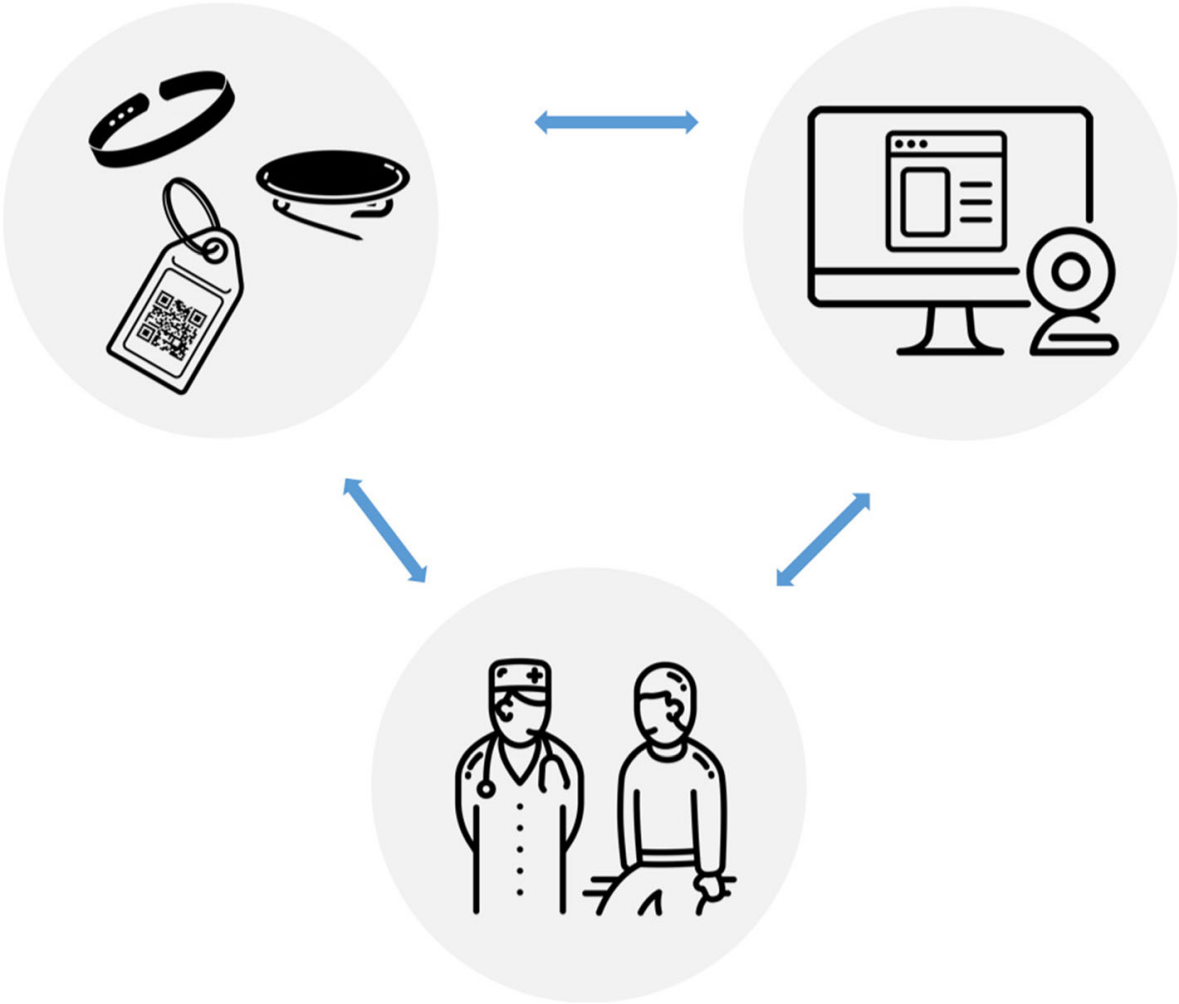
Illustration of Tiny task as inspiration for a physical reminder (created using ‘Sketch’ [https://www.sketchapp.com/])

In each assessment session, the executive researcher (SS) presented an overview of 1) the web-based e-learning module (interactive ‘clickable’ wireframe) (see Fig. 2); 2) an envisioned scenario in which an oncologist interacts with the chatbot (a mockup of an automated chat conversation with a digital ‘coach’) (Fig. 3) and 3) a sketch of the physical reminders (Fig. 4).

Building on the specified user requirements (see Table 2), the presented prototypes of the e-learning and chatbot contained simulations of the following interactions (see for more details Table 3): 1) the possibility to assess information giving skills before, during and after training, and receive performance feedback; 2) the possibility to personalize the learning experience and set individual learning goals; 3) the possibility to acquire information about a topic of interest; 4) the possibility to practice newly acquired information; 5) receiving reminders of learning goals during training and daily practice; 6) the possibility to ask for personal (professional) coaching. The presentation of these simulated interactions between the user and the tool, made it easier for the participating hemato-oncologists to judge the advantages and disadvantages of the suggested prototypes.

**Table 3:** Description of the main features of the e-learning and chatbot

Feature	Description^a^
1: Entry test	Participants had to perform an ‘entry-test’ [9] comprising the video-recording of a consultation with one of their own patients [14]. This video-recording had to be sent to peers, who provided immediate feedback [15]. This task had to be performed in the e-learning module as well as Chatbot.
2. Personalization	In the e-learning; the content and some assignments could be tailored to participants’ learning needs (e.g. to self-select a module [4], to obtain access to supportive literature [7]). Moreover, the chatbot offered additional choices to personalize the training experience such as whether the participant preferred to receive instructional material as text or video [10], or how much time the participant wanted to spend on this specific session.
3. Information presentation	The content of the instructional material was the same in both the e-learning and chatbot and comprised brief text, an animation and video fragments of consultations [7, 10, 11]. However, in the Chatbot this material was presented more interactive, whereas in the e-learning it was presented more static (scrolling and clicking).
4. Assignments	The e-learning contained assignments such as reviewing video-recorded consultations (their own or of colleagues) and leaving a verbal message in response to an utterance of a videotaped patient [12]. The chatbot only contained the assignment to leave a verbal message (to not to have too many repetitive tasks during the testing session).
5. Reminder	In the e-learning, an email appeared to remind the participant of a personal learning goal [18]. The chatbot provided a notification on the participants cell phone, as a reminder.
6. Expert coaching	The e-learning module contained a menu-option facilitating consultation with a communication expert [21]. In de chatbot module, the option was offered to consult a communication expert for example after having received a too low grade for an assignment.

^a^The numbers in brackets refer to the requirements described in Table 2

##### Analysis

Both assessment sessions were transcribed verbatim and a thematic analysis was conducted using inductive coding. Transcripts were read multiple times for familiarity and independently coded by two coders (SS, ES). Initial coding attached basic surface-level labels to data. Both researchers subsequently discussed differences in interpretations until consensus was reached between the two coders. Next, codes addressing similar themes were collapsed to form subthemes. Findings were presented at an INSTRUCT expert-group meeting for critical reflection.

##### Results

Based on the presentation, participants in both *user group assessment* sessions favored the conventional e-learning over the chatbot. In their view, an e-learning would make it easier to focus on and dedicate time to the acquisition of skills, as compared to the more easily accessible, but fragmented approach via a chat-bot. However, participants considered individual differences in this preference likely, depending on age and familiarity with the use of (social) media on smartphones. The possibility to compose a personal learning track (feature 1,2 and 4), based on an initial assessment, was greatly appreciated, although it was noted that this possibility might withhold some individuals from receiving the modules they would actually need. Elements to enhance the transfer of skills to daily practice (features 2 and 5) were considered more suitable for a chatbot as compared to e-learning. The idea of somehow using reminders (feature 5) was generally well-received, although none of the participants saw any added value in the tangible physical reminders. Importantly, participants resisted too many different information channels. All agreed that any training, regardless of the medium, should focus as much as possible on video-recordings of own, real-life consultations (feature 2) and facilitate personal feedback from a communication expert (feature 1 and 6). Based on these comments, we further pursued the development of an interactive prototype of both the e-learning and chatbot, but not of the physical reminders.

### Individual user-evaluations of two prototypes

After several iterations of developing interactive prototypes by the lead researcher (SS) based on input of the aforementioned group evaluation and in close collaboration with expert members of the INSTRUCT learning format panel, we subsequently conducted one-hour *individual user-testing* sessions.

#### Participants and procedure

The sample comprised five junior oncologists in training at the Amsterdam University Medical Center/University of Amsterdam (age range 31–37; 3 women), who were recruited via the hemato-oncologist involved in the INSTRUCT group (MK). Each test-session started with the e-learning module, followed by the chatbot, and ended with evaluative questions addressing their appreciation of (aspects) of the training tools. Participants were explicitly instructed to pretend using each of the six simulated interactions offered by the tool (see Table 3) and perform two assignments for both tools while thinking aloud during these tasks. The two simulated assignments comprised 1) the evaluation and/or annotation of a video-recording of an actual consultation (of their own or a colleague as part of a pre-post training test, or an instructional fragment) and 2) the recording of their verbal reaction in response to a video-recorded utterance of a patient. Following a think-out-loud protocol [56], the executive researcher (SS) probed for clarification and suggestions and asked open-ended questions during the testing session concerning the usability and feasibility of the interactive prototypes. All sessions were video-recorded and transcribed to be able to systematically observe how participants used the training tools [57, 58]. A test protocol ensured consistency across individual testing sessions.

#### Analyses

The executive researcher (SS) performed an initial analysis by grouping all remarks of participating physicians according to one of six pre-defined themes (remarks about the digital training tools overall; the format of instructions; the assignments; the format of feedback on assignments; assessment and about the comparison e-learning versus chatbot) and per theme as either positive, negative, or ‘other’. This overview was independently summarized by two researchers (ES, CD) and subsequently discussed to reach consensus about main findings.

#### Results

Results revealed that, of the five participants, four expressed a willingness to use a digital training tool to acquire information-provision skills. They appreciated the innovative approach and saw merit in the possibilities offered by the technology to perform the training in their own time and attuned to their own learning needs.

*I’m a big proponent of e-learning because everybody can learn things in their own way. I think it is a great additional, … additional tool to improve your skills in your own time. (#1, female)*

*A greater variety of ways to study would be wonderful. That you can just … we can just learn something and that your phone kinda does it for you … that you only have to read a piece of text or watch a short film … that information is just provided in the form of a text message, that is of course brilliant. –because we are so much used to that. That is much nicer than a text book. (#2, male)*

Participants questioned the reliability and value of a formal assessment such as an entry test on the basis of the limited number of consultations that would be assessed, the assumed lack of clear criteria for rating communication skills, and the risk that trainees would then only upload their ‘best’ consultations.

*There is also a chance that physicians will chose patients who are ‘easy’ … that is also my experience. You are likely to choose a conversation that’s running smoothly; you are not going to use your worst conversations for this [e-learning]. (#2, male)*

*. Assessing everything isn’t feasible,, but you’re doing it based on one video. It remains, well … a subjective assessment. Suppose you could upload more videos. Then it would be less about this one patient were things maybe didn’t go as smooth. Yet, this would come with the disadvantage of being very time-intense. (#3, female)*

Regarding the information presentation, participants appreciated the combination of text, educational animation, and illustrative/exemplary video fragments of skills as offered in both the e-learning and chatbot.

*Generally, I appreciate to watch short films. Otherwise, it tends to be a lot of text that doesn’t stick [with you]. (#1, female)*

*[in response to the opportunity to watch short exemplary/prototypical films:] “it is very useful to have examples of conversation techniques. You can always read about it, but you just have to do it. (#2, male).*

*It’s great that it looks appealing, some visual cues are nice. (#4, male)*

Regarding the illustrative video fragments, some participants expressed a wish to see both positive and negative examples of skills. References to background papers, provided as hyperlinks, or brief summaries thereof were welcomed. Participants liked the ‘tone of voice’ to be informal and personal (e.g. your consultation vs the consultation) yet not too informal (in particular with regard to the chatbot).

Concerning the assignments, the functionality of annotating fragments of video-recorded consultations was evaluated positively.

*Its’ great. You learn from yourself and others; by receiving feedback from others.(#5, female)*

It was recommended to make the assignment concrete, not overly easy and to announce that feedback would be provided to enhance motivation. All five test participants valued the option to upload and respond to their own video-recordings and those of peers. They considered this to be novel, motivating, valuable, insightful, and better than actually attending someone else’s consultation.

*I would find it irritating if someone was sitting next to me [in clinic] and watching all my movements. Thus, I appreciate the videos. It’s a little bit of hurdle, but gives you lots of insights; cause you are also re-watching it by yourself. As opposed to having someone sit next to you [in clinic], then you would have to discuss everything right away –otherwise you have already forgotten what you had discussed with the patients. Thus, I think this is a very useful aspect of it. (#3, female)*

*Yeah, I think it is very good to upload something, cause you have obviously thought about it yourself; and then you would like to hear what someone else might think about it. I think that’s great. (#4, male)*

Critical comments concerned emotional concerns or practical barriers (time consuming, technical problems) for making a recording. Some saw more value in discussing video’s in a (small) group.

*It makes you quite vulnerable to have the conversation video-taped; especially if you’re not used to it. I think that [this aspect] could cause some hesitation. But by now, we’ve done it so many times that it became routine. (#5, female)*

The following suggestions were given to improve this video-feedback feature: involve experts, have several rather than one person provide feedback and guarantee privacy, which includes a) permission of patients; b) restrictions in the time that video’s are accessible for feedback and c) explicit agreements about respecting privacy.

The assignment to leave a verbal message in response to an utterance of a video-recorded patient was considered “fun”, stimulating and relevant for the transfer of skills to clinical practice.

*Well that you yourself –that is pretty funny – [laughing]. I like the idea that you … you obviously miss the body language, but words … you hear what somebody else would say … better than writing it down.(#2, male)*

*Oh wonderful, that is actually funny:* Interviewer: Would you use this feature yourself? *Yes, certainly, in this manner. I would totally do that..(#3, female)*

*Yes it is nice; it’s about communication techniques and you sit silently in front of a computer by yourself. No, that is nice … I think it’s a nice experiment, that I would encourage. (#5, female)*

As a downside, it was noted that the presence of others (e.g. colleagues in the room) may be a barrier to use this functionality. Therefore, providing the option to (also) type a response should be considered.

Both the e-learning and chatbot offered the feature of generating reminders. Some saw the added value (# 5: “it would help me”) whereas others would fear message overload, in particular when these would appear on their mobile phone.

Participants were somewhat skeptical about the lack of face-to-face feedback which they considered critical to acquire communication skills. Therefore, the option to receive expert coaching was well-received.

*I think nobody knows that this is available. I’ve been thinking more often about how to do it. One person is obviously different from another, thus I think it would be very nice if you had the opportunity to ask someone to provide input once in a while. –but that never occurred to me (#3, female)*

Additionally, some concerns were voiced about privacy issues regarding the use of video-recordings of consultations. Importantly, it was noted that the use of training tools needed to be formally coordinated (e.g. clear deadlines, facilitation by coordinator, criteria for ‘success’, accreditation points).

Four out of the five test participants favored the chatbot over the e-learning. The following positive reactions were voiced about the chatbot: refreshing, faster and a more informal form of communication, doesn’t feel as work, low threshold to use this, convenient, might more easily integrate in daily clinical routine.

*It’s just faster; you have more of a feeling … with e-learning it always feels like such movies take 5 minutes [gestures wildly], but that the essence/take-home message could have been told on only one minute. Here you can chose if you want to watch a short movie including an example / … e-learning gives you that feeling that you don’t have to listen to a full lecture; I can learn my way, and actually gain more. (#1, female)*

*I think it’s fun; it’s a nice idea’ it’s more modern … I learned more from this training than from the previous. Thus, I think it works very well. Yet, I foresee some practical issues: I imagine it being difficult to motivate everybody to install the app and actually do this training with each other. I think this will be difficult, but if you do it in a specific period of time, it would be possible I think. (#4, male)*

*You have to make it practical, right! … otherwise, you have to go back to your computer and log-in again. However, if this is on your phone and you receive reminders to work on a certain topic … yeah, that’s very accessible (#5, female)*

On a more critical note, one participant felt that the chatbot was rather in the lead whereas she would like to be more in control herself. Some participants questioned the value of a chatbot for watching video’s or reading longer texts and felt they would be more easily distracted when using a chatbot as compared to an e-learning.

*I think it’s difficult to watch a movie, if it’s on your phone. Extensive text is even more difficult I believe; too much information. … You also showed a link to more information, this seems less handy to read on your phone. Yet, the way [of having a chatbot] appeals to me a lot. (#1, female)*

Two participants suggested to combine the e-learning with a chatbot, by providing learning modules via a more conventional e-learning and a digital coach to help them in their daily practice.

*It would be interesting to combine this [chatbot] with the e-learning: That based on your answers, you will get support from the chatbot. For example, “I can see that these are topics you’re struggling with, would you like me to tell you more about ‘tailoring’ (#4, male)*

## Discussion

This paper describes the first three phases of the CeHRes Roadmap [39] for the user-centred design of a digital training tool aimed at supporting oncologists’ acquisition of information giving skills in the context of oncology treatment. Digital training tools for communication skills training seem promising because they offer busy oncologists the opportunity to gain knowledge about and to practice their communication skills at their own time and pace, at a location of their choice and attuned to their own learning needs. Yet, to the best of our knowledge, no such trainings are currently available.

Participating oncologists acknowledged the relevance of CST and responded positively to our proposed digital training formats.

We provide insight into the issues that influence oncologists’ decisions to eventually use such a training tool.

Based on literature, our starting point of building on the principles of experiential practice [47] and reflective learning [48], consultation of the users in the inquiry phase of the development process [14], and on expert opinion in the value specification phase, essential elements to consider for the digital training format were *personalization* and *interactivity.*

### Personalization

In our prototypes we applied different strategies to offer users a personalized experience. First, by asking oncologists to inform us what skills they want to learn regarding treatment information provision, we could start with content attuned to their needs. Subsequently, we allowed users to pick and choose how they would learn by offering a non-linear approach. That is, they could skip or choose particular content and thus map their own learning path. Of note, this runs the risk that learners skip content that would actually be relevant to them. Therefore, any personalized tool should eventually also incorporate a progress monitoring system to assess whether, despite personalization, the tool is delivering the desired results - in our case, whether information giving skills actually improve. To this end, our proposed tool included the option of a pre- and post-test. To further personalize the learning experience, we also tried to tap into the users’ learning style and preference by offering the option to choose from text, video, and animation. This feature was much appreciated. Moreover, we started with developing an e-learning chatbot and physical reminders to see which learning environment would fit oncologists best. The idea of physical reminders was quickly discarded, as none of the participants appreciated the concept. Additionally, we encountered clear differences between users in their appreciation of the e-learning versus conversational chatbot user-interface. Purely from a personalization perspective it might be recommended to integrate both interfaces in the digital training, so the presentation format can be user-defined. For optimal personalization, age factors, including experience with technology need to be taken into account. We found oncologists in training to be more interested in the chatbot than more senior oncologists. By offering both the e-learning and chatbot in the digital training, both senior and younger clinicians might be reached.

### Interactivity

The need for interactivity is in line with a review which concludes that learners greatly value (digital) courses that allow to enter into a dialogue with a course tutor, fellow students, or a virtual tutorial and to obtain ongoing feedback [59]. Oncologists are no exception in this regard. The real time chat function in our chatbot is an example of a functionality aimed at interactive dialogue.

The importance of interactivity to support peer-feedback is intrinsically connected to the premises of experiential and reflective learning [47, 48] and to the Professional and Scholar roles of the CanMeds model, which is nowadays the leading educational model underlying many medical curricula in the world [60]. Participating oncologists underscored the importance of peer-feedback as a relevant feature of any training tool and suggested themselves the use of videos of clinical consultations. Video-based learning, as proposed in our prototypes, by sharing and commenting on video-recordings of clinical performance has been found to be effective [61, 62]. Importantly, for actual implementation of such a feature, privacy issues need to be taken well care of to safeguard the privacy of patients as well as learners.

In addition to peer-feedback, many oncologists indicated the wish to also receive personal feedback from a communication expert. Expert feedback may be relevant because peers may be biased in a positive direction, guided by not wanting to be too critical in their feedback to preserve the interpersonal relationship [63]. To accommodate oncologists’ wish, we included the option to consult a communication expert in our tool, which was highly appreciated. However, this raises the question whether it is possible to fully digitalize a communication skills training. A blended learning approach, combining the advantages of technology with traditional teaching strategies, may therefore be the key to uptake in this context. Blended learning integrates asynchronous e-learning, which is independent of time and space, and synchronous on-site learning, which is facilitated at a fixed time by an educator [64]. Online learning platforms can offer the opportunity for also the communication expert to review and annotate the work of students at their own time and place. Online video-feedback is feasible and effective [61, 65]. This way, the advantages of both approaches can be combined. This is supported by the finding that participants from blended learning programs especially emphasized the value of the face-to-face components [31, 32, 66].

### The CeHres roapmap

A particular challenge of this project was to create a tool that was engaging enough to capture the interest of busy oncologists. We therefore deliberately chose an iterative design process, such as the CeHRes roadmap, to create as many opportunities as possible to check that the tools we were developing would align with their needs and preferences. This stepwise approach, comprising interviews, focus groups, user assessments, and individual user testing, was helpful in generating valuable feedback from the users. A lesson learned is that the use of small steps also has some disadvantages. We noticed that participants became progressively more enthusiastic about the tools, the further developed they became. In a first presentation, the possibilities and functionalities of the foreseen training tools are still rather abstract and may therefore not come across as very attractive. Hence, it is challenging for a developer to keep users engaged for further testing. Another lesson learned is that one should not strive for ‘one size fits all’. Personalization may be a way to also engage those who are initially reluctant, such as more senior clinicians. It implies that to accommodate and thus reach as many individuals as possible with a communication skills training, different training formats should be offered alongside each other. In hindsight, our decision to focus on oncologists in training because they seemed more interested in computer based training may have been a missed opportunity to learn how to engage senior clinicians.

In our foreseen digital training we focused on the skill of information-provision. Information-provision is particularly challenging in the oncology setting, although specialists in other disciplines may be confronted with similar issues. Oncology consultations are generally characterized by high information density due to the complexity of the life threatening diagnoses, the numerous treatment options that are often available, and their associated risk of serious side-effects. Importantly, this information is likely to cause emotional distress in patients. For all of these reasons, the importance of high quality communication skills is generally well recognized in the oncology setting, as illustrated by published guidelines [11]. Despite room for improvement, communication skills trainings for oncology clinicians have been widely implemented in the last decades [67].

In view of this context, medical oncologists might be relatively prone to be involved in the development of a tool for communication skills, as compared to clinicians in medical specialties with less attention for this competency. Additionally, a digital training targeted at information-provision skills of oncologists will be particularly useful as a refresher training to maintain or further improve their previously acquired skills. For other medical specialists, it may be more relevant to start with developing basic communication skills. This most likely will require more traditional face-to-face communication skills training.

Integrating stakeholder involvement in the development process maximizes acceptability, potential effectiveness and thereby eventual implementation. However, it is challenging to integrate expert knowledge and scientific evidence in the process of attempting to include and accommodate subjective opinions and experiences from end-users. We struggled with this tension. We performed a literature search to create an inventory of the potential of an online intervention for communication skills training and involved experts in the ‘learning content’ and ‘learning format’ group. Hence, members of the research team were knowledgeable about the state of the art. Yet, at the same time, we did not want to bias the participants too much by introducing this knowledge, out of fear to restrict the richness of their input regarding desired features of the tool and consequently to create functionalities they themselves would not have proposed. As a consequence, our eventual prototypes may be considered relatively conventional.

To address this tension, future developers may consider following the procedure as suggested by O’Brien and colleagues [68]. They propose, as a stage 1 in an iterative user-centered design process, to compile the evidence base for the issue addressed by the foreseen tool, preferably, albeit not exclusively, by systematic reviews. Next, this evidence is recorded as a list of ‘evidence statements’ which inform the aims and content of subsequent co-design sessions. If evidence needs to be compiled, this approach can be labor and time intensive, and thus it would take a considerable proportion of the time-line of a project. Where recently conducted, high quality systematic reviews exist, these can be used to develop evidence statements to inform the co-design process [68]. The introduction of ‘evidence statements’ would certainly be attractive to clinicians, as they are used to and value the use of systematic reviews as an important pillar of evidence based medicine.

### Limitations and future directions

The following limitations need to be considered. Although we applied a user-centered approach, by choosing to use the CeHRes framework, this does not mean that the ideas were fully co-created with the end-users. Participant samples in each of the different phases were relatively homogeneous and small and it was rather demanding to involve professionals over a longer period of time. Although it is generally known that co-design asks for considerable involvement of the participants, we nevertheless again emphasize the effort that is required to engage medical professionals in such a process As a result, limited conclusions can be drawn. However, because in this early phase of development we were validating design decisions prior to a formal evaluation, the qualitative data has been useful to inform the development of our prototypes. Future studies, covering phase 4 (operationalization) and 5 (evaluation) of the CeHRes roadmap, will need to progressively include more participants and outcomes have to be measured more rigorously, encompassing rigorous behavioural observation and standardized assessment instruments (e.g. the System Usibility Scale), based on technology acceptance models such as the Unified Theory of Acceptance and Use of Technology (UTAUT), to assess the tools’ usability, uptake and effectiveness [69, 70]. Finally, our prototypes of training tools focused on one particular skill only. That is, on the tailoring of information to patient characteristics such as their need for information or level of understanding. Tailoring of information is an important skill encompassing various sub tasks as illustrated in Table 1. Nevertheless, other information skills are equally important in discussing cancer treatment information, such as structuring of the information and demonstrating empathy. We decided to narrow the focus down to tailoring, for the purpose of developing a prototype. A future tool may very well also address other relevant communication skills.

Technology acceptance models [59, 70] predict that if oncologists would perceive the characteristics of the digital tools, such as their easy availability, as advantages over a more traditional face-to-face training format, they will be more likely to perceive the tools as useful and therefore more inclined to accept them. Issues of acceptance, adoption and adherence as well as the effectiveness of the tools in improving oncologist’s information-giving skills have to be tested in summative evaluation studies, as part of the last phase of the CeHRes roadmap (see Fig. 1) [39]. Only rigorous evaluation studies, using the full spectrum of qualitative and quantitative evaluation methods, will be able to fill the gap between the postulated benefits of digital training tools for communication skills and actual outcomes [71]. On a last note, we acknowledge that even if we succeed in developing a tool that is accepted by oncologists, i.e. perceived as useful and easy to use, and that has been proven effective, that is succeeds in improving oncologists’ information giving skills, contextual factors such as support from superiors, accreditation points, and costs will determine the success of its implementation.

## Conclusion

In conclusion, extensive development work was conducted in collaboration with multiple health care professionals to iteratively develop two innovative prototypes of digital tools that would appropriately engage oncologists in learning effective information giving skills. We expect that spending time on the design process will pay off later, as such that we were able to gather insightful data from users that informed the eventual prototypes. The prototypes that were developed upon both literature as well as users’ needs, were well appreciated and thus provide a solid basis for further development and testing in the operationalization phase, involving the introduction and use in actual practice [39].

## Data Availability

The original data can be retrieved via the corresponding author.

## Abbreviations

CanMeds: Canadian Medical Education Directives for Specialists
CeHRes: Center for eHealth Research
CST: Communication skills training
HCP: Health Care Professional
UTAUT: Unified Theory of Acceptance and Use of Technology
